# A note on the relation of anisotropic peak broadening with lattice symmetry in powder diffraction

**DOI:** 10.1107/S2053273325003134

**Published:** 2025-04-24

**Authors:** Piotr Fabrykiewicz

**Affiliations:** a Institute of Crystallography, RWTH Aachen University, Jägerstr. 17-19, 52066 Aachen, Germany; b Jülich Centre for Neutron Science at Heinz Maier-Leibnitz Zentrum, Forschungszentrum Jülich GmbH, Lichtenbergstr. 1, 85747 Garching, Germany; Institute of Crystallography - CNR, Bari, Italy

**Keywords:** Rietveld refinement, pseudosymmetry, microstructures

## Abstract

A bridge is established between the Gregorkiewitz & Boschetti [*Acta Cryst.* (2024), A**80**, 439–445] and Stephens [*J. Appl. Cryst.* (1999), **32**, 281–289] formalisms of anisotropic peak broadening in powder diffraction. The paper by Gregorkiewitz & Boschetti presented formulas describing position shifts of low-symmetry peaks due to different lattice relaxation schemes. Anisotropic peak broadening caused by lattice relaxation can be parameterized by the variance of slightly dispersed peaks’ positions. The calculated variances are compared with formulas from the widely used phenomenological model of anisotropic peak broadening by Stephens. Specific relations between anisotropic peak broadening parameters can be a hint of a possible unresolved peak splitting due to lattice symmetry lowering.

## Abstract 2

The variance of 1/*d*^2^_*hkl*_ positions for different lattice relaxation schemes is calculated and compared with literature formulas for anisotropic peak broadening in powder diffraction.

The paper by Gregorkiewitz & Boschetti (2024 ▸) presented formulas for the 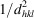 position of *hkl* contributions to the same (*hkl*) peak in a powder diffraction pattern considering six minimal lattice symmetry relaxation schemes: (1) cubic to tetragonal, (2) cubic to rhombohedral, (3) hexagonal to orthorhombic/monoclinic, (3*a*) hexagonal to orthorhombic, (4) tetragonal to orthorhombic, (5) orthorhombic to monoclinic, (6) monoclinic to anorthic (triclinic). Based on these formulas I calculated the variances 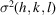 of the 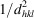 values of the *hkl* contributions. For all relaxation schemes the variances 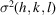 are expressed as fourth-order polynomials in *h*, *k*, *l* indices: 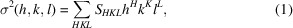
with 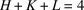. Symmetry restrictions for each Laue class for 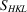 coefficients were given by Popa (1998 ▸). A phenomenological model of anisotropic peak broadening (Stephens, 1999 ▸; Popa, 1998 ▸) assumes that each crystallite in a powder sample is in general triclinic and that only average lattice constants over the whole sample fulfil restrictions of a given lattice symmetry. In this approach 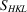 coefficients are linear combinations of covariance matrix elements, which describe a lattice constant distribution in the polycrystalline sample (see Stephens, 1999 ▸). For each of the lattice relaxation schemes (1–6) listed above, the barycentre shift 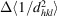, the variance 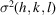 of 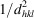 and the relations between 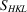 parameters are given in Table 1 ▸. The effective peak widths calculated in a Rietveld refinement depend on the instrumental resolution function, on 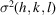 and on possible other effects, *e.g.* crystallite size effects *etc*. Without specifying the peakshape function one can say that the total peak width is monotonic with 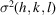.

The anisotropic peak broadening method is often used to improve the Rietveld refinement, but the values of the Stephens parameters 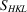 are not always discussed. The equalities between 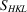 parameters in Table 1 ▸ included in square brackets, *e.g.*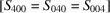, are identical to those given by Stephens (1999 ▸). The relations between 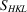 parameters included in two neighbouring brackets, *e.g.*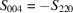 (see cubic to tetragonal in Table 1 ▸) are additional restrictions due to the specific lattice relaxation scheme and they were not provided by Gregorkiewitz & Boschetti (2024 ▸). The equations in Table 1 ▸ form a bridge between the Gregorkiewitz & Boschetti (2024 ▸) and Stephens (1999 ▸) formalisms, giving the values of the 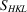 parameters in terms of lattice parameter increments. One has to keep in mind that definitions of anisotropic peak broadening parameters used in Rietveld refinement software do not always agree with the definitions from the Stephens (1999 ▸) paper. For a quantitative analysis of the 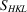 parameters one should refer to the documentation of the specific Rietveld refinement program in use.

Leineweber (2017 ▸) showed a relationship between anisotropic peak broadening parameters and the strain tensor within symmetry-related low-symmetry domain states. Applying his general formula [see equation 10 of Leineweber (2017 ▸)] to the cubic to tetragonal lattice relaxation scheme leads to the same relationship between 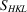 parameters as given for this scheme in Table 1 ▸ [see equation 17 of Leineweber (2017 ▸)].

The study of Leineweber (2017 ▸) points out that the high-symmetry model with anisotropic peak broadening parameters and the low-symmetry model with isotropic peak broadening frequently yield in Rietveld refinements similar agreement factors. Therefore the choice of the proper model should be made in combination with possibly available additional information. Statistically relevant non-zero values of 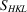 parameters fulfilling the relations given in Table 1 ▸ obtained in Rietveld refinement of powder diffraction data can be a signature of a possible unresolved peak splitting due to lattice symmetry lowering. It should be possible to implement an automatic check of this relationship in Rietveld analysis software, which will alert the user to when there are grounds to consider a low-symmetry lattice model. Users could also manually constrain 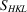 parameters to follow equalities given in Table 1 ▸.

Additional remark: in the Gregorkiewitz & Boschetti (2024 ▸) supporting information I found an error in the equations of barycentre shift for relaxation from cubic to rhombohedral and from orthorhombic to monoclinic. Instead of 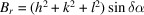 and 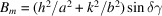, it should be 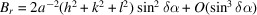 and 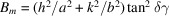, respectively.

## Figures and Tables

**Table 1:** Number of split peaks for general (*hkl*), values of barycentre shift 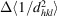 within linear approximation, variances 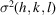 of dispersed 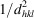 positions within quadratic approximation and relations between anisotropic peak broadening parameters 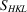 as defined by Stephens (1999 ▸) for all relaxation schemes discussed by Gregorkiewitz & Boschetti (2024 ▸)

1. Cubic to tetragonal (3 peaks) 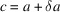
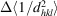 = 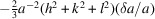
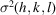 = 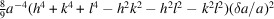
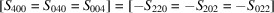 = 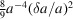
(*HHH*), locally narrowest; (*H*00), locally broadest

2. Cubic to rhombohedral (4 peaks) 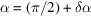
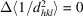
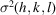 = 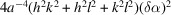
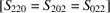 = 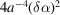 and 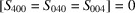
(*H*00), locally narrowest; (*HHH*), locally broadest

3. Hexagonal to orthorhombic/monoclinic (6 peaks)
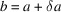 and 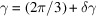
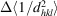 = 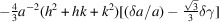
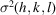 = 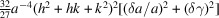
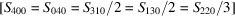 = 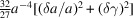
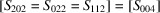 = 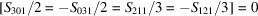
(00*L*), locally narrowest; (*HKL*), locally broadest

3*a*. Hexagonal to orthorhombic (3 peaks)
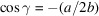 and 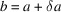
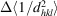 = 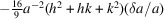
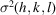 = 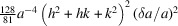
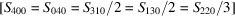 = 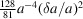
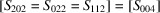 = 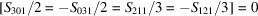
(00*L*), locally narrowest; (*HKL*), locally broadest

4. Tetragonal to orthorhombic (2 peaks) 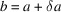
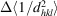 = 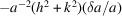
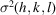 = 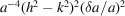
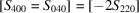 = 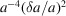
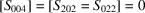
(*HHL*), locally narrowest; (*H*0*L*), locally broadest

5. Orthorhombic to monoclinic (2 peaks) 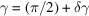
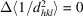
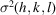 = 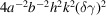
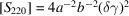
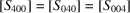 = 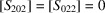
(*H*0*L*), (0*KL*), locally narrowest; (*HKL*), locally broadest

6. Monoclinic to anorthic (triclinic) (2 peaks)
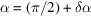 and 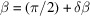
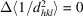
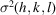 = 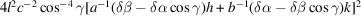
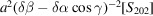 = 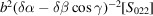 = 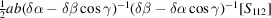 = 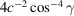
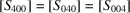 = 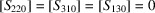
(*HK*0) and (00*L*), locally narrowest
